# Remarks on the Various Forms of Quackery

**Published:** 1815-01

**Authors:** Geo. Stevens

**Affiliations:** Yorkshire


*For the Medical and Physical Journal.*

### *Remarks on the various Forms of Quackery;* by Mr. G. Stevens.

BEING an old bachelor, very much at leisure, part of my time is killed at cards, and part in reading periodical publications: among the rest, the Medical and Physical Journal serves me for conversation with our village doctor, when I can get him to a rubber. When it is our turn to set out, I have often remarked how much more lookers-on see of the game than those who are actually at play. This has suggested suggested to me the idea, that, if you will admit a paper from an unprofessional man, your readers may meet with hints which may not occur to your usual correspondents. A passage I lately met with in Baron Grimm's Memoirs, has furnished me with a subject.

"The most celebrated practitioner that ever appeared at Paris, (says that entertaining writer,) was a private in the French guards. His only remedy was a *tisane of hay* boiled in water. He treated his patients *en bêtes*, and he judged rightly on the subject. This decoction of hay soon placed him in a situation to give good dry forage to two horses which were harnessed to a good carriage, in which he rode to visit his patients, whilst the then *docteur regent* of the faculty was making his rounds on foot through the *boue de Paris.* The faculty soon after made a complaint to the Marechal de Biron, to oblige this hay-doctor to lay down his equipage, and reserve all his forage for his patients."

When I hear the learned part of your profession exclaim against quackery, I often wish to know which of them is guiltless, to cast the first stone. Was this private in the guards the only practitioner whose patients were taking an equally innocent tisane, and how many of those whose remedies were more powerful were really succeeding better, and, after all, are we certain that there is no efficacy in a decoction of hay? Ought there not, therefore, to be certain lines fixed between the quackery of regulars and the quackery of professed quacks?

Nothing is more common than for a medical man, when he reads in the paper how much better my Lord or my Lord Duke has found himself by the attendance of Dr. ——, or Sir ——, to remark—"a good puff!"—"a good piece of quackery!" This is indeed now so well understood, that the officers of the revenue, I am informed, always demand the customary duty of advertisement on such paragraphs. But there is another species of quackery, in my opinion, much less excuseable, and much more artful. The trick I allude to is the publication of half what the author means to tell the world. Thus, one writer informs us, that to distinguish certain complaints requires great discernment, long practice, a particular habit of attending to these subjects; in short, it is not difficult to see that he means—"You must apply to me, and I do not wish to say too much; some people may think lest I should render them as wise as myself; but the truth is, I know no more than you, and therefore do not chuse to expose myself."—One or both reasons must be the true one, and whichever it may be the quackery is the same.

The

The next species of regular quackery is the publication of a single volume on any subject, with the promise of compleating the work hereafter. On this occasion we have always an enormous deal of learning, ten thousand suggestions about the importance of certain questions, and the number of experiments and observations which the author found it necessary to make before he could satisfy himself in certain intricacies. The last thing in all these cases, and what the reader is anxiously looking for, is the treatment; but, when he comes to the end, he finds the author has not entirely made up his mind on some points, or that his various occupations have not allowed him leisure to complete this part of the work to his satisfaction; he promises, however, that it shall soon appear: in the mean while, whom can the reader consult so well as this learned and experienced writer?

The last kind of professional quackery which I shall notice, is one which it is astonishing to me that the college has not hitherto noticed in their own body; this is the courage with which physicians, usually the youngest of the order, attempt to lecture on all the various branches of the art. As they grow older they seem to be more modest, and to satisfy themselves with one branch only. This, Messrs. Editors, is a crying evil. Every man who undertakes to teach is presumed to have devoted his time exclusively to the science of his lectures; by these means he is not only more intimately acquainted with his subject, but learns also the best mode of teaching it. Can this be expected from any one in two such sciences as medicine and chemistry? The first considered, since the days of Hippocrates, as too much for a man's life, and the second improving so rapidly every hour, that the best chemists, whose whole time is engaged in such studies, not unfrequently learn the fallacy of those lessons which they delivered only a week before. I am told, that, when the college see any thing unbecoming in the conduct of their members, they usually send them an admonition: have they ever sent such admonitions to unfledged doctors, who undertake to teach at an age when their own education must be very incomplete, or who venture to lecture on more than one branch? Till these things are attended to, I think it as well to be cautious how the regulars, as they call themselves, talk of quackery.

*Yorkshire;* GEO. STEVENS.

